# Beyond Local Gating: Toward Network-State-Dependent Neuromodulation

**DOI:** 10.3390/bioengineering13101151

**Published:** 2026-10-02

**Authors:** Paolo Belardinelli, Elena Mongiardini, Sonia Turrini, Alessio Avenanti

**Affiliations:** 1 Department of Psychology and Neuroscience—DIPseN, University of Trento, 38068 Rovereto, TN, Italy; elena.mongiardini@unitn.it; 2 Department of Neurology & Stroke, University of Tübingen, 72076 Tübingen, Germany; 3 Hertie-Institute for Clinical Brain Research, University of Tübingen, 72076 Tübingen, Germany; 4 Center for Studies and Research in Cognitive Neuroscience, Department of Psychology “Renzo Canestrari”, Alma Mater Studiorum Università di Bologna, Cesena Campus, 47521 Cesena, FC, Italyalessio.avenanti@unibo.it (A.A.); 5 Centro de Investigación en Neuropsicología y Neurociencias Cognitivas, Universidad Católica del Maule, Talca 3460000, Chile

**Keywords:** non-invasive brain stimulation (NIBS), cortico-cortical paired associative stimulation (ccPAS), network-state-triggered neuromodulation, adaptive network-guided neuromodulation

## Abstract

Brain stimulation is progressively evolving from anatomically targeted interventions toward individualized, adaptive, and network-oriented approaches. Recent developments in brain-state-dependent stimulation have demonstrated that neuromodulation can be optimized by synchronizing stimulation with ongoing neural activity, while adaptive cortico-cortical paired associative stimulation (ccPAS) has extended this principle to distributed cortical networks. However, current approaches continue to define brain state primarily through local electrophysiological features, such as oscillatory phase or power, even though ccPAS is specifically designed to modify communication between interconnected cortical regions. In this opinion paper, we argue that the endogenous variable guiding ccPAS should instead describe the instantaneous physiological state of the targeted cortico-cortical pathway. We propose that online source-space functional connectivity provides a biologically meaningful and technically feasible estimate of this pathway state, thereby enabling a new framework of network-state-dependent neuromodulation. As illustrative applications, we consider pathways spanning different levels of experimental characterization, from established sensorimotor interactions to the less-characterized ventral premotor cortex (PMv)-posterior superior temporal sulcus (pSTS) pathway within the action-observation network (AON). This distinction highlights that the proposed framework is pathway-general, while its experimental implementation requires pathway-specific characterization of stimulation parameters and appropriate physiological readouts. In this framework, resting-state EEG is rapidly reconstructed in source space to estimate pathway connectivity, which is subsequently used to personalize stimulation targets and guide state-dependent ccPAS of a functionally defined cortico-cortical circuit. Finally, we discuss the technological prerequisites, methodological challenges, biological rationale, and open scientific questions that must be addressed before pathway-state-guided neuromodulation can be experimentally validated. We argue that redefining brain state as the instantaneous physiological state of the communication pathway targeted by stimulation may represent the next conceptual step in brain stimulation.

## 1. From Anatomical Targeting to Network-State-Dependent Stimulation

Non-invasive brain stimulation (NIBS), particularly transcranial magnetic stimulation (TMS), is a powerful tool for probing and modulating human brain function in both basic neuroscience and clinical research. Building on conventional single-site TMS, cortico-cortical paired associative stimulation (ccPAS) delivers precisely timed magnetic pulses to two interconnected cortical regions with the aim of inducing long-lasting modifications of their functional interactions through Hebbian-like spike-timing-dependent plasticity (STDP) mechanisms [1,2,3,4,5,6,7,8]. To date, ccPAS has been applied across several functional systems, most extensively within motor and visuomotor networks. In these domains, protocols targeting premotor–motor, parietal–motor, supplementary motor–motor, cerebellar–motor, and interhemispheric pathways have induced sustained changes in corticospinal excitability and in paired-pulse TMS, EEG, and TMS-EEG measures of pathway strength and interregional coupling [3,4,9,10,11,12,13,14,15,16,17,18,19,20]. These physiological effects have been accompanied by selective changes in motor performance, action imitation, and inhibitory control, depending on the targeted pathway [9,21,22,23,24,25,26]. More recently, ccPAS has been extended to perceptual, metacognitive, and executive domains [27,28,29,30,31,32,33,34], establishing it as an increasingly versatile and powerful network-based approach for causally probing and modifying specific cortico-cortical pathways. Overall, available evidence indicates that ccPAS can induce pathway-, direction-, and timing-dependent LTP- or LTD-like changes in cortical excitability and interregional coupling that persist beyond the stimulation period and selectively influence functions supported by the targeted network. Consistent with this view, a systematic review and meta-analysis found reliable, medium-sized effects on both neurophysiological and behavioral outcomes across cortical systems [2]. A summary of the key methodological parameters governing ccPAS protocols—including stimulation order, inter-stimulus interval, pulse intensity, and pairing frequency/number—is provided in Box 1.

Over the past two decades, NIBS has progressively evolved from anatomically defined interventions toward increasingly individualized and adaptive stimulation strategies. Early TMS and ccPAS protocols targeted predefined cortical regions based on structural landmarks or stereotactic coordinates, assuming that stimulation of a specific anatomical location would produce predictable physiological effects (e.g., M1 in TMS-EEG or PMv–M1_hand_ in ccPAS [13,23,35,36,37]). More recently, advances in neuroimaging and neurophysiology have enabled functional targeting, whereby stimulation sites are selected according to task-related activity or each individual’s intrinsic functional organization (e.g., [38]).

A key conceptual advance in TMS research has been the increased recognition of state-dependency effects. A large body of work has shown that the neural activation state present at the moment of stimulation—a state that can be set by what the participant is perceiving or doing—determines the physiological and behavioral consequences of TMS [39,40,41,42,43,44,45,46]. Applied to ccPAS, this principle implies that the behavioral or perceptual state engaging a pathway during stimulation steers which functionally specific subpopulation undergoes plasticity. Consistent with this, pairing stimulation with a viewed motion direction yields direction-specific gains along the V5/MT+-V1/V2 pathway [47], preconditioning specific visuomotor associations biases plasticity within the PMv–M1_hand_ pathway [48,49], and pairing with a specific facial expression selectively strengthens emotion-tuned pSTS → V1/V2 circuits. These observations reinforce the idea that the physiological substrate of ccPAS is the dynamic state of the pathway itself, not merely the activity of its individual cortical nodes. Crucially, in all these behavioral approaches, the plasticity-permissive state is never observed directly. It is *inferred* from the manipulation used to induce it, on the assumption that a given task, at a given instant, engages a given neuronal population, while the neural state itself remains a black box, accessed only through the behavior presumed to drive it. This raises a natural question: rather than inferring the state behaviorally, could stimulation be timed to a state *read directly* from ongoing brain activity?

Brain-state-dependent stimulation *takes exactly this step*, delivering TMS pulses only when ongoing neural activity satisfies predefined physiological criteria, such as oscillatory phase, oscillatory power, or cortical excitability state [50,51,52,53,54,55,56,57,58,59]. These studies demonstrated that the efficacy of neuromodulation depends not only on *where* stimulation is delivered, but also on *when*. More recently, Ermolova et al. [60] extended this principle to ccPAS by triggering paired-pulse stimulation according to the instantaneous sensorimotor μ-rhythm recorded simultaneously over the two stimulated motor cortices. Although the triggering criterion was jointly defined by the oscillatory states of two groups of electrodes around C3 and C4 (namely, targeting the mu-alpha oscillation in both sites, the mutual triggering conditions were trough-trough, trough-positive peak, positive peak-trough, random phase stimulation), it remained fundamentally a local electrophysiological descriptor rather than a direct measure of the functional interaction between them. Consequently, the physiological substrate targeted by ccPAS, the cortico-cortical pathway itself, was not used to guide stimulation.

A further advance has recently been provided by Jovellar et al. [61], who demonstrated the feasibility of real-time brain-state-coupled ccPAS in a fronto-parietal cognitive network, extending adaptive paired associative stimulation beyond the motor system. In their protocol, however, stimulation timing was gated exclusively by the positive theta phase estimated at a single site, the dorsomedial prefrontal cortex, with the paired parietal pulse following at a fixed 10 ms interval regardless of its own local state. Although the intervention successfully reconfigured fronto-parietal connectivity (decreased gamma- and increased alpha-band coupling), this connectivity was assessed only as a post hoc outcome measure rather than used to inform the triggering algorithm. This work thus establishes that adaptive ccPAS can be extended to distributed cortical networks and represents an important milestone toward individualized neuromodulation, yet the endogenous variable defining stimulation timing remains a local descriptor—a single-site oscillatory phase rather than a joint or pathway-level measure of the interaction between the two stimulated regions.

Historically, then, brain-state-dependent neuromodulation opened this box only partway: it reads the neural state directly, but as a local property of the stimulated regions taken individually. For an intervention designed specifically to modify cortico-cortical communication, such a local readout is itself only a proxy for the quantity that matters, the state of the *interaction* between the two stimulated regions. We propose that the relevant endogenous variable should instead describe the instantaneous physiological state of the communication pathway linking them.

A first step toward this perspective was provided by [53], who demonstrated that the single-trial phase-locking value computed online between two linear combinations of EEG electrodes can serve as a network-based triggering variable for TMS. Building on these developments, we propose that the next conceptual evolution of adaptive neuromodulation is network-state-dependent stimulation, in which online source-space functional connectivity is used to estimate the physiological state of a targeted cortico-cortical pathway before and during stimulation. Within this framework, connectivity is no longer viewed simply as an outcome measure, but as the endogenous state variable used to personalize stimulation targets and guide ccPAS delivery.

**Figure 1:**
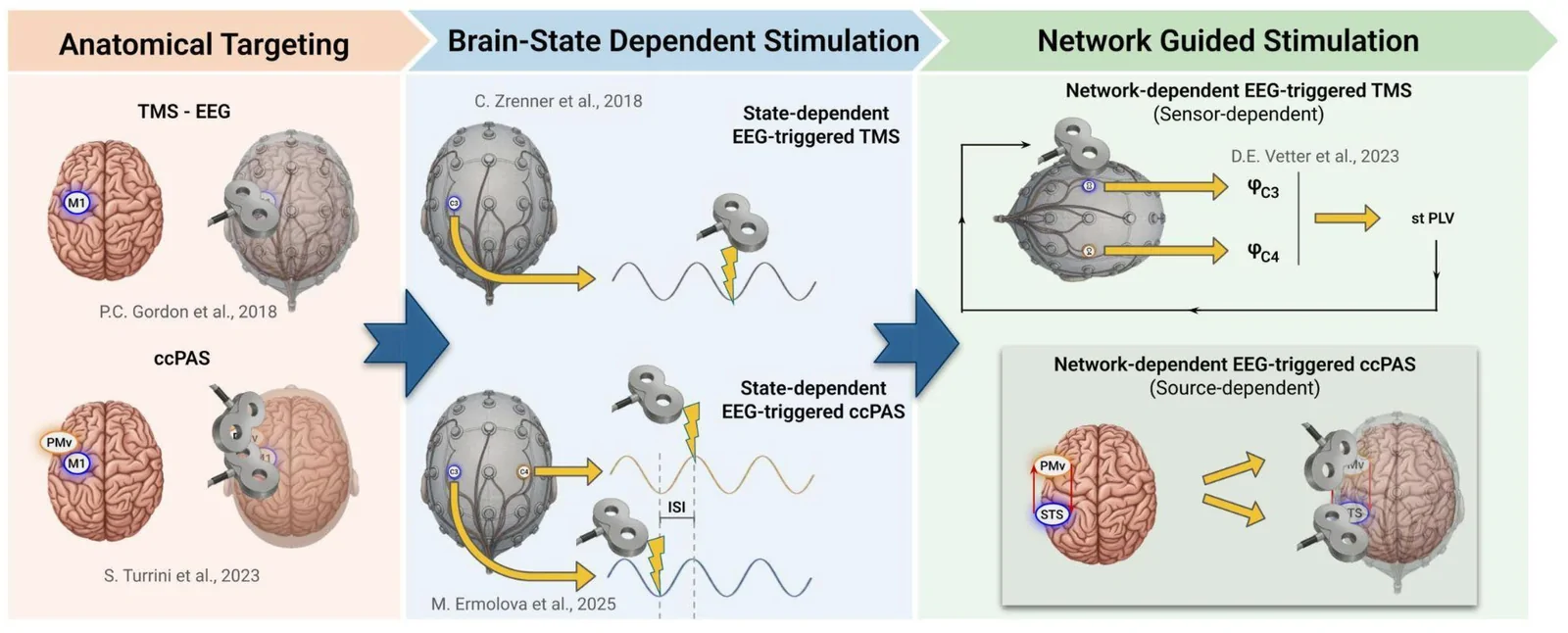
From anatomical targeting to network-guided stimulation. Conceptual evolution of non-invasive brain stimulation strategies, illustrated across three successive stages. *Anatomical Targeting* (**left panel**): classical TMS-EEG and ccPAS protocols relying on fixed anatomical targets, identified through structural landmarks or standard stereotactic coordinates (e.g., M1 for TMS-EEG [35]; PMv–M1_hand_ for ccPAS, [13,23]). *Brain-State Dependent Stimulation* (**central panel**): protocols in which stimulation delivery is gated by the instantaneous oscillatory state recorded via EEG [55], or according to the instantaneous oscillatory state of two cortical sites in EEG-triggered ccPAS [60]. *Network Guided Stimulation* (**right panel**): the approach proposed in this work, in which stimulation is guided by the ongoing pathway connectivity between distributed regions. Top: an example of network-dependent EEG-triggered TMS based on the single-trial phase-locking value (st PLV) computed between the phases of two linear combinations of electrodes around C3 and C4 [53]. Bottom: conceptual schematic of the network-dependent EEG-triggered ccPAS proposed here, in which online connectivity between two functionally interconnected cortical regions (e.g., PMv and pSTS) is used as the triggering criterion for stimulation delivery. AI was used to generate some portions of the figures. The authors have reviewed and edited the output and take full responsibility for the content of this publication.

To make this proposal experimentally concrete, we consider an initial implementation in which pathway state is operationalized as non-zero-lag phase synchronization between the source-reconstructed activity of the two targeted cortical regions, quantified using the imaginary phase-locking value (iPLV) within a predefined frequency band and sliding temporal window [62,63,64]. Individual resting-state data are used to establish subject-specific connectivity thresholds, and subsequent ccPAS volleys are delivered when the online connectivity estimate satisfies the predefined state criterion, subject to stimulation and signal-quality constraints. This provides a fixed, a priori triggering rule with which the central hypothesis—whether the ongoing connectivity state of a pathway predicts its susceptibility to ccPAS-induced plasticity—can be directly tested. Figure 1 summarizes this conceptual progression—from anatomically targeted stimulation, through functional targeting and brain-state-dependent neuromodulation, to adaptive ccPAS and the pathway-oriented network-state-dependent framework proposed here—in which online source-space connectivity between two interconnected cortical regions (e.g., PMv and pSTS) is used to determine stimulation delivery.

This framework therefore shifts the central question from *when stimulation should be delivered* to *what physiological property of the targeted pathway should define the brain state itself.*

Box 1 **Stimulation parameters governing ccPAS-induced plasticity.**

 ccPAS entails the repeated stimulation of two cortical sites, using two independent TMS devices and coils, with a precise interstimulus interval (ISI) between the paired pulses. In addition, the stimulation is delivered at a predetermined global frequency, and the overall duration of the protocol depends on the number of paired pulses administered. Therefore, the parameters that need to be optimized when designing a ccPAS protocol are (1) the stimulation intensity at the two targeted sites; (2) the ISI between the two pulses of each pair; (3) the frequency of the paired stimulation; and (4) the relative order of the two pulses within each pair. While these parameters were originally established for motor-system applications, they generalize to any two-node cortico-cortical pathway, including functional hubs of non-motor networks [2]. Below, we consider each of these parameters individually.

 (1) **Stimulation intensity:** The relative intensity of the two pulses shapes the sign and magnitude of the conditioning effect, as shown for the premotor–motor pathway, where the influence of premotor stimulation over M1 excitability shifts from inhibitory to facilitatory depending on intensity [65,66]. For motor areas, the gold standard remains referencing the resting motor threshold (rMT), and indeed the vast majority of published ccPAS studies targeting motor regions adapt stimulation intensity individually based on this measure, even when the second, non-motor site of the targeted pathway lacks an equivalent physiological readout. Notably, even when targeting non-motor regions, many ccPAS studies still reference the rMT as a proxy for individual cortical excitability, despite the stimulated site being anatomically and functionally distinct from M1. For visual cortex stimulation, some studies have adapted stimulation intensity based on the phosphene threshold [47], whereas other studies have adopted fixed intensities (e.g., [27]). Alternatively, intensity can in principle be approximated via scalp-to-cortex distance scaling [67] or, more recently, via individualized electric-field modeling [68,69,70,71]. A further, newer alternative is the closed-loop optimization of stimulation parameters based on real-time TMS-evoked EEG responses [72]. However, none of these approaches have yet been applied to ccPAS protocols.

 (2) **ISI:** The temporal gap between the two pulses must fall within a physiologically effective STDP window; when suboptimal, plasticity effects are reduced [73] or abolished entirely [74,75]. In motor-system ccPAS, ISIs have typically been derived from dual-coil effective-connectivity studies using MEP amplitude as a direct readout [13,21,76]. For pathways lacking such a readout, ISIs have instead been estimated from TMS-EEG conduction-timing measurements [27] or from the oscillatory period of the pathway’s dominant rhythm, as in the gamma-band-calibrated ISI used for interhemispheric visual-motion areas [73].

 (3) **Frequency and number of paired pulses:** Most ccPAS protocols to date have used low repetition rates in the infraslow range (0.1–0.25 Hz) and about 100–200 pulse pairs, values largely inherited from early motor-system studies [4,14,76,77]. The choice of an infraslow frequency, and 0.1 Hz in particular, is not arbitrary: earlier work has shown that repetitive TMS delivered to a single cortical hotspot at 0.1 Hz does not, by itself, induce any lasting potentiation or depression of cortical excitability, even when applied continuously for up to an hour [78]. This provides researchers with the confidence that any plastic changes observed following a ccPAS protocol can be attributed to the paired stimulation of the two targeted sites, rather than to the repeated stimulation of either site in isolation. Nevertheless, it should be noted that neither the pairing frequency nor the total number of pulse pairs has been systematically or directly investigated as a parameter in its own right within ccPAS research: to date, no study has directly compared protocols of different durations or delivered at different (rhythmic or non-rhythmic) frequencies, on the resulting magnitude or direction of the induced plasticity. As such, this remains an important open parameter for future methodological investigation.

 (4) **Stimulation order.** Determining which region receives the first versus second paired pulse is arguably the most critical parameter in ccPAS protocols, as it dictates the direction of the induced plasticity according to Hebbian STDP principles [1,79,80,81,82]. Consistent with this, the vast majority of ccPAS studies published to date have used the reversed stimulation order (i.e., stimulating area B before area A, rather than area A before area B) as a control condition, precisely because reversing pulse order has been consistently shown to yield different, and often opposite, effects on connectivity and cortical excitability [2,14,23,24,49,76,83]. This reinforces that stimulation order is not a minor methodological detail but a defining feature of the protocol. However, establishing the appropriate stimulation order is considerably more straightforward for some pathways than for others. For monosynaptic, well-characterized circuits, such as the extensively studied PMv–M1 pathway, established neurophysiological and neuroanatomical [84,85,86,87], as well as human effective connectivity data (e.g., from dual-coil TMS studies demonstrating short-latency, direction-specific influences of PMv over M1; [65,66,88,89,90,91]) provide a solid empirical basis for inferring which area should be conditioned first. In these cases, the choice of stimulation order can be directly grounded in prior physiological characterization of the pathway. By contrast, when ccPAS is applied to large-scale, distributed cortical networks involving polysynaptic connections (e.g., prefronto-parietal or default-mode/task-positive networks), determining the appropriate stimulation order becomes considerably less straightforward. In these cases, our understanding of the relationship between the two areas is often derived largely from correlational neuroimaging evidence (e.g., fMRI functional connectivity or coherence between EEG signals). The stimulation order must therefore be inferred from theoretical models of network function, whereby one region is hypothesized to act as the primary driver or originator of activity and the other as the receiver. Importantly, this very ambiguity is also what makes ccPAS a particularly valuable tool for investigating polysynaptic networks. Where correlational imaging methods can only describe that two regions are functionally coupled, ccPAS offers a way to experimentally manipulate the directionality of that coupling and thereby test competing theoretical accounts of network organization. By selectively strengthening connectivity in one direction (A-to-B) versus the reverse (B-to-A), it becomes possible to establish whether a specific cognitive function or behavioral capacity depends on directed influence from area A to area B, from area B to area A, or on the reciprocal balance between the two, a question that purely correlational approaches cannot resolve. This is illustrated, for instance, by [74], who showed that ccPAS applied over the dorsolateral prefrontal cortex—posterior parietal cortex (DLPFC-PPC) network produced opposite effects on DLPFC cortical excitability depending on stimulation order (DLPFC-to-PPC versus PPC-to-DLPFC), and by [38], who used ccPAS to test the hypothesized anticorrelated relationship between the default mode network (DMN) and the task-positive network (TPN), demonstrating that manipulating connectivity in one direction (TPN-to-DMN) could partially reverse this anticorrelation. Both studies illustrate how ccPAS can move beyond descriptive accounts of functional connectivity to provide causal evidence about the directionality and functional relevance of large-scale network interactions.

## 2. Why Pathway Connectivity Should Matter

This is the question we take up here. If the endogenous variable guiding ccPAS is intended to describe the physiological state of the targeted cortico-cortical pathway, we must identify a *physiological property* that captures the state of the *interaction* between the two regions, rather than of either region in isolation. We propose that *source-space functional connectivity* is a biologically plausible candidate, because it reflects the moment-to-moment coordination between the very neuronal populations whose interaction ccPAS is designed to modify. Throughout this paper, we distinguish the physiological construct of *pathway state* from its operational measurement. We define *pathway state* as the physiological condition of the interaction between the two cortical populations targeted by ccPAS over a finite temporal window preceding stimulation. For the implementation proposed here, we operationalize this latent state as the strength of non-zero-lag phase synchronization between source-reconstructed activity in the two targeted regions, quantified using the iPLV within a predefined frequency band and sliding temporal window. Candidate high- and low-connectivity states are defined by comparing the resulting iPLV estimate with subject-specific thresholds established a priori from resting-state data. Thus, pathway state does not refer generically to any measure of statistical dependence between two regions; iPLV constitutes the specific observable proposed here for testing whether the ongoing phase-coupling state of the targeted pathway predicts susceptibility to ccPAS-induced plasticity. Other connectivity measures, including amplitude-based or directed estimators, represent alternative operational definitions whose relative predictive value remains an empirical question.

This proposal differs from the state-dependent approaches described above primarily in what is considered the relevant state variable. Previous approaches have either imposed a favorable brain state exogenously by engaging participants in a specific behavioral task during stimulation [47,49], or estimated brain state online as a local property of the individual stimulated regions [60,61]. By contrast, our framework asks whether the endogenous state of the interaction itself—arising spontaneously and measured online without behavioral manipulation—can serve as the relevant state variable, identifying from ongoing neural activity the moments at which the pathway is most susceptible to Hebbian-like STDP. Whether such an internally emerging network state predicts the induction of plasticity as effectively as experimentally imposed or locally defined brain states has, to the best of our knowledge, not yet been directly investigated. This represents the specific conceptual advance proposed by our framework.

Several studies have demonstrated that ccPAS can modulate corticospinal excitability, oscillatory dynamics, and electrophysiological measures of cortico-cortical coupling [4,14,60,75]. Nevertheless, substantial inter-individual variability remains one of the major limitations of ccPAS [12,92], suggesting that the responsiveness of a cortico-cortical pathway depends on physiological factors that are not captured by current stimulation paradigms. These observations motivate the search for endogenous variables capable of characterizing the instantaneous physiological state of the pathway before and during stimulation.

Why should functional connectivity represent pathway state? From a biological perspective, several complementary theoretical frameworks point toward this possibility. Hebbian-like STDP posits that long-term synaptic modification depends on the coordinated activation of interconnected neuronal populations [1]. Likewise, theories of communication-through-coherence propose that information transfer is dynamically regulated by transient synchronization between distributed cortical regions rather than by the activity of isolated cortical areas [93,94,95,96,97]. Together with the growing body of evidence supporting state-dependent neuromodulation [98,99], these concepts suggest that the susceptibility of a cortico-cortical pathway to plasticity may depend on its ongoing functional coupling rather than solely on the local activity of its constituent regions.

Importantly, this hypothesis remains untested. The online connectivity estimate of the targeted pathway has not yet been demonstrated to predict the efficacy of ccPAS. Accordingly, the framework proposed here should not be regarded as an empirically validated stimulation protocol, but as a testable experimental hypothesis. Its central prediction is that ccPAS delivered during predefined pathway-connectivity states will produce systematically different plastic effects from stimulation delivered during other states or without state-dependent triggering. Failure to observe such state-dependent differences would argue against that specific operationalization of pathway state as a determinant of ccPAS-induced plasticity. Thus, we propose that the functional connectivity of the targeted pathway should itself become the object of investigation, not merely as an outcome measure of stimulation, but as a candidate endogenous state variable capable of explaining, and ultimately guiding, stimulation-induced plasticity. The decisive form of this question—whether the online connectivity estimate of the targeted pathway *predicts* the induced plasticity, rather than merely changing as a consequence of it—is one we return to in Section 5.

Until recently, this question could not be addressed experimentally. Advances in EEG source reconstruction, parcel-wise source modeling, leakage-robust connectivity estimation, and computational efficiency now make it increasingly feasible to estimate the source-space functional connectivity of a targeted cortico-cortical pathway within the timescale of a single experimental session [100]. At the same time, recent perspective papers have highlighted the transition from open-loop toward adaptive brain stimulation and emphasized the need for endogenous physiological variables capable of guiding individualized neuromodulation [56,101]. While oscillatory phase, oscillatory power, and functional connectivity have all been proposed as candidate physiological descriptors, the specific role of online source-space functional connectivity as an estimate of pathway state for adaptive ccPAS has received remarkably little attention. We argue that recent methodological advances now make this hypothesis both technically feasible and experimentally testable.

If pathway connectivity is to become the endogenous variable guiding adaptive ccPAS, the next question is whether it can be estimated rapidly, reliably, and robustly enough to inform stimulation within a single experimental session. The following section examines the technological advances that now make this possibility increasingly realistic.

## 3. A Framework for Network-State-Dependent ccPAS

If the functional connectivity of a targeted cortico-cortical pathway can be regarded as a physiological descriptor of its instantaneous pathway state, the next challenge is to incorporate this information into the control logic of network-state-dependent neuromodulation. We propose a framework in which online estimates of source-space functional connectivity are no longer treated simply as descriptive measures of network organization or as post hoc outcome measures but instead become endogenous variables that actively guide stimulation delivery in real time—the quantity used to determine *when* each TMS paired-pulse volley is delivered—on the basis of the instantaneous state of the interaction between the two stimulated regions.

The framework can be understood as two stages of increased ambition. The first, which we refer to as *connectivity-informed stimulation*, can already be implemented using existing methodologies. A short resting-state EEG recording is acquired before the stimulation session, reconstructed into source space, and used to estimate parcel-level functional connectivity. The resulting individualized network architecture identifies the most relevant cortico-cortical pathway and provides subject-specific stimulation targets for the subsequent ccPAS intervention. An analogous logic of connectivity-based personalization has already been applied using functional magnetic resonance imaging (fMRI) to select ccPAS targets [38,102]; what we propose here is an EEG-based, source-space equivalent obtainable within the same session, immediately before stimulation. In this first stage, connectivity informs stimulation planning but does not directly determine stimulation timing.

The second stage, which constitutes the principal conceptual contribution of this paper, is *network-state-triggered ccPAS*. Here, online estimates of source-space functional connectivity are continuously obtained during the experimental session and used to estimate the instantaneous physiological state of the targeted cortico-cortical pathway for each stimulation volley, on a moment-to-moment basis, so that each paired-pulse volley is delivered only when the pathway occupies a candidate plasticity-permissive state. This extends the logic of oscillatory brain-state-dependent stimulation, in which pulses are gated by an instantaneous local feature such as phase or power, to the pathway level, where the gating variable is the ongoing coupling estimate between the two stimulated regions. In this stage, the candidate state is defined a priori, for example, epochs of high versus low pathway connectivity, with individualized thresholds derived from the initial resting-state recording. The aim is correspondingly circumscribed, to test whether triggering ccPAS on such states alters the magnitude or direction of the induced plasticity relative to state-uninformed or randomly timed stimulation, and it is achievable with existing technology.

**Figure 2:**
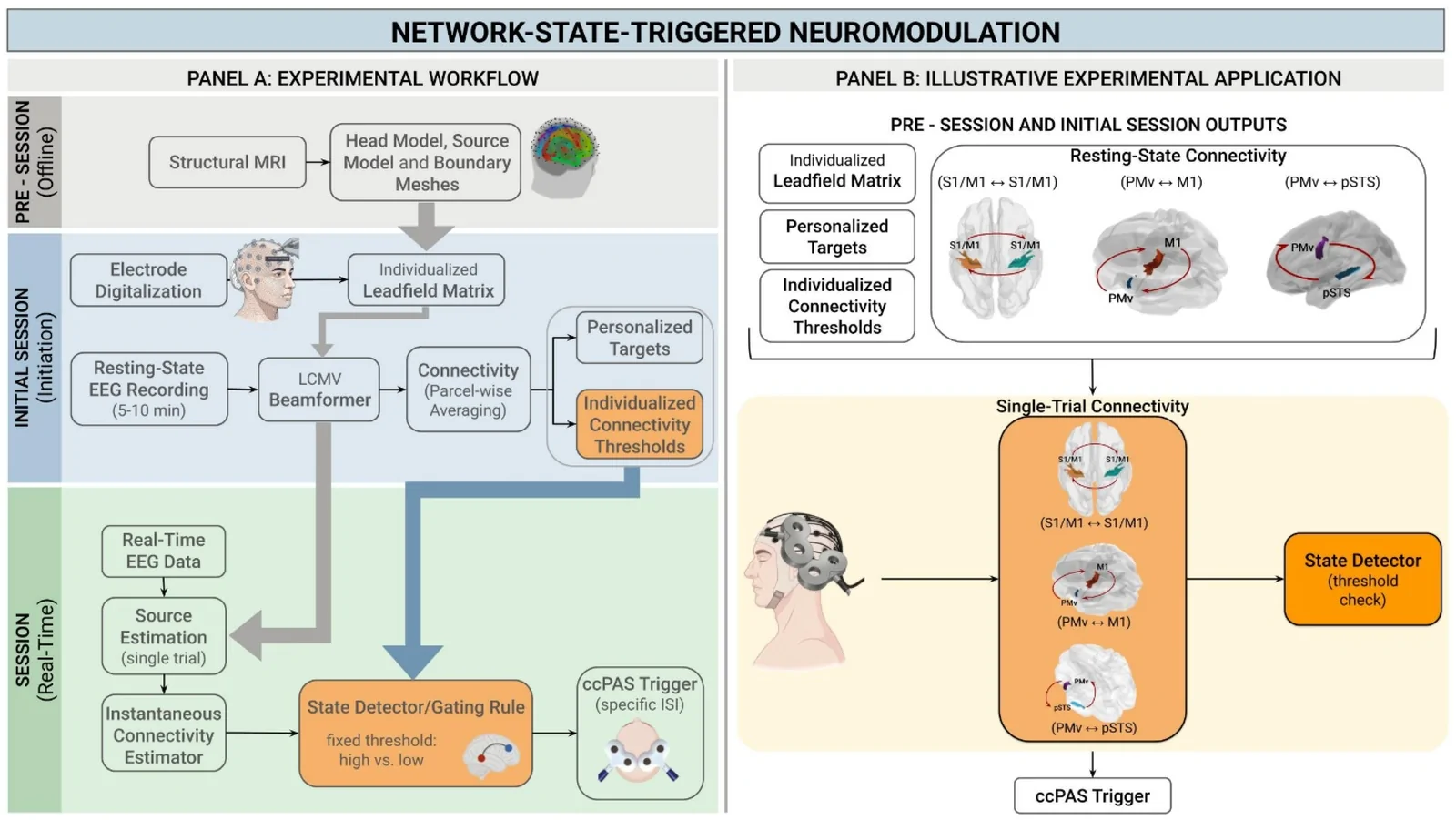
Network–state–triggered neuromodulation: proposed workflow and illustrative application. (**A**) Experimental workflow. *Pre-session (offline)*: individual structural MRI and EEG-electrode digitization are used to build a subject-specific head and source model and the corresponding individualized leadfield matrix. *Initial session (initiation)*: a short resting-state EEG recording (5–10 min) is projected into source space with an LCMV beamformer, and parcel-wise functional connectivity is estimated to (i) identify personalized stimulation targets and (ii) define individualized connectivity thresholds that specify the candidate plasticity-permissive state for that participant. *Session (real-time)*: real-time EEG is used to reconstruct source activity and to estimate the online connectivity estimate of the targeted pathway; whenever this estimate satisfies the predefined threshold, a ccPAS volley is delivered at the pathway-specific inter-stimulus interval. Stimulation is thus gated by the instantaneous pathway state according to a fixed, a priori rule, without online updating of the gating criterion. (**B**) Illustrative experimental application. Three candidate applications spanning different levels of experimental characterization: the interhemispheric right–left S1/M1_hand_ and the M1_hand_–PMv pathway as comparatively well-characterized sensorimotor examples suitable for initial validation, and the PMv–pSTS pathway as an exploratory extension to a less-characterized distributed non-motor interaction. For each pathway considered, pre- and initial-session outputs provide the individualized leadfield, the personalized targets, and the resting-state connectivity thresholds. During the real-time session, single-trial connectivity is continuously estimated and compared against these thresholds, triggering ccPAS when the pathway occupies the candidate state. In the panel, both the right–left S1/M1_hand_ and PMv–M1_hand_ pathways that can be used for initial validation and the PMv–pSTS pathway are reported. AI was used to generate some portions of the figures. The authors have reviewed and edited the output and take full responsibility for the content of this publication.

This framework is illustrated in Figure 2, where Panel A depicts the proposed general workflow, from offline personalization to the real-time triggering, and Panel B applies the same logic to the action observation network. For initial experimental validation, the framework is most appropriately tested in comparatively well-characterized sensorimotor pathways, where stimulation parameters, directional interactions, and independent physiological readouts are more readily available. Examples include PMv–M1_hand_ and interhemispheric sensorimotor pathways, for which established physiological measures can provide outcomes independent of the EEG connectivity metric used for triggering [88,89,90,91,103,104,105,106,107,108,109,110,111]. Such systems reduce uncertainty related to pathway anatomy, stimulation parameters, and outcome measurement, allowing an initial validation study to focus specifically on whether pre-stimulation pathway state predicts the magnitude or direction of ccPAS-induced plasticity. By contrast, the PMv–pSTS pathway considered below serves as a deliberately more exploratory example. These features would reduce uncertainty related to pathway anatomy, stimulation order, inter-stimulus interval, and outcome measurement, allowing an initial validation study to focus specifically on whether the pre-stimulation connectivity state predicts the magnitude or direction of ccPAS-induced plasticity. The PMv–pSTS pathway, which is thought to rely substantially on indirect, polysynaptic routes involving the inferior parietal cortex [112,113,114] serves a different purpose: it provides an illustrative example of how pathway-state-dependent stimulation might subsequently be extended to less-characterized, distributed cortical interactions. PMv and pSTS are key nodes of the action-observation network (AON), linking higher-order visual representations of observed actions with parietal and premotor sensorimotor processes involved in action perception and prediction [112,115,116,117]. Precisely because ccPAS has not yet been applied to this pathway, its stimulation order and effective inter-stimulus interval would need to be independently established before pathway-state-dependent stimulation could be implemented. As for any ccPAS protocol, the inter-stimulus interval and stimulation order would need to be calibrated to the effective communication latency and dominant direction of this specific pathway, as previously done for the pSTS-V1/V2 loop from TMS-EEG timing estimates [27,118], rather than assumed a priori. A critical feature of this initial validation is the separation between the variable used to define pathway state and the measure used to quantify induced plasticity. Functional connectivity would serve as the triggering variable, whereas the primary outcome should preferentially be an independent physiological readout. In well-characterized sensorimotor pathways, such as PMv–M1_hand_ and interhemispheric S1/M1_hand_, induced plasticity can be assessed through physiological measures that are independent of the EEG connectivity metric used for triggering, including MEP amplitude, dual-coil TMS measures of effective connectivity, or TMS-EEG measures, depending on the pathway and stimulation protocol; behavioral measures can provide complementary outcomes. For PMv–M1_hand_ specifically, MEP amplitude provides a particularly direct and established physiological readout. Connectivity itself may additionally be assessed as a secondary outcome, but using it as both the triggering variable and the sole measure of plasticity could introduce regression-to-the-mean effects and correlated measurement error. Demonstrating that connectivity-defined pathway state predicts changes in an independent physiological measure would therefore provide a more stringent test of the central hypothesis.

**Figure 3:**
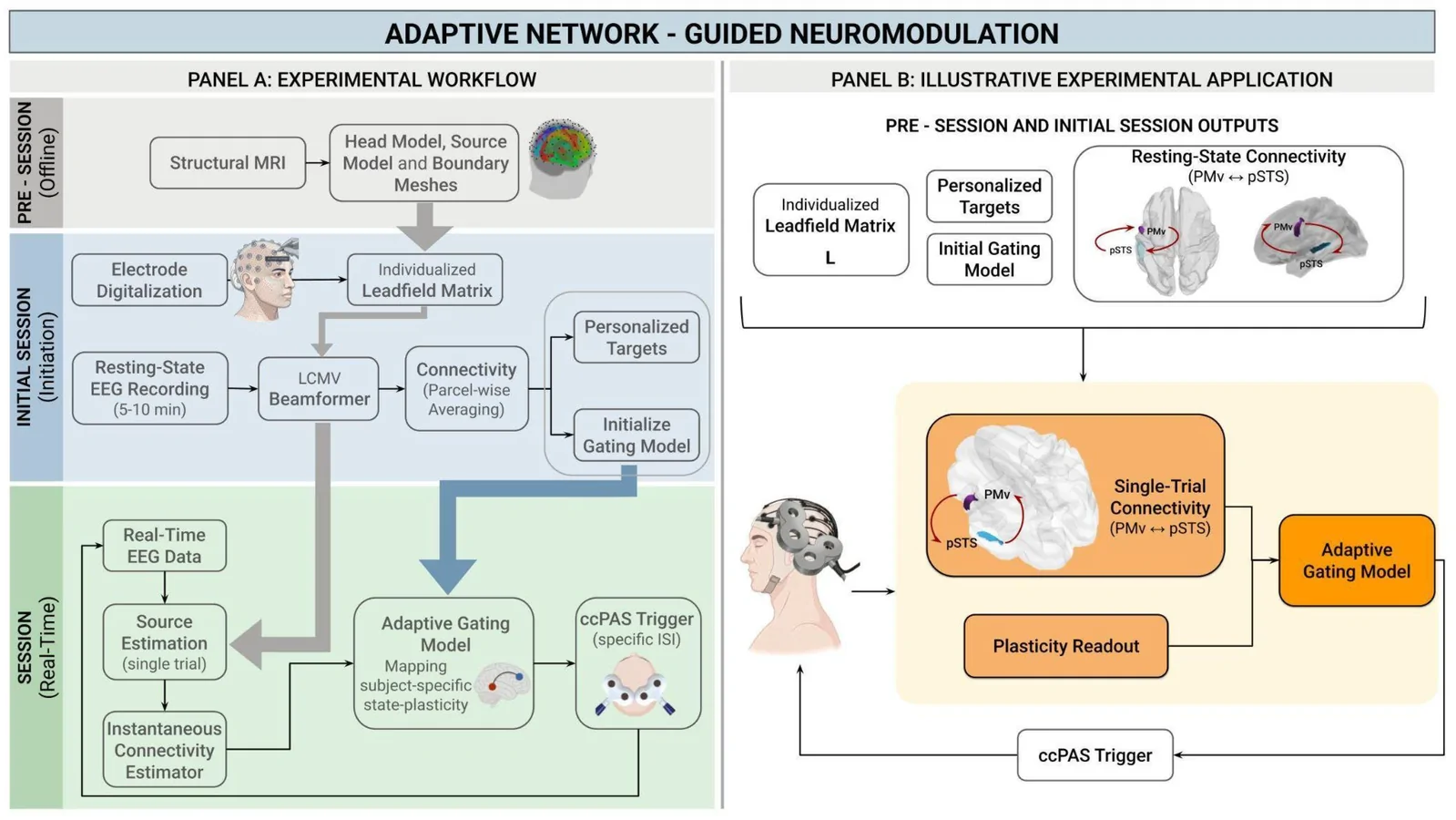
Adaptive network–guided neuromodulation: proposed experimental workflow and illustrative application. This scheme extends the workflow of Figure 2 by closing the loop not only on pathway *state* but on the plasticity that stimulation *induces.* (**A**) Experimental workflow. *Pre-session (offline)*: individual MRI and EEG electrode digitization are used to construct a subject-specific head and source model, from which an individualized leadfield matrix is derived. *Initial session (initiation)*: a short resting-state EEG recording is projected into source space using an LCMV beamformer, and parcel-wise connectivity is estimated to identify personalized stimulation targets and to initialize the subject-specific gating model. *Session (real-time)*: during the real-time session, source activity and pathway connectivity would be continuously estimated and provided to an outcome-adaptive gating model together with a physiological readout of stimulation-induced plasticity. If a sufficiently rapid and specific plasticity readout becomes available, the model could progressively learn the subject-specific relationship between pathway state at stimulation and the ensuing physiological change and use this mapping to update subsequent triggering decisions. Because no such validated online plasticity readout is currently available, this configuration is presented as a future, conditional extension rather than a currently implementable protocol. (**B**) Illustrative experimental application. Pre-session and initial-session outputs are derived from the individualized head model and from resting-state connectivity between a selected region pair (here, PMv and pSTS). During the real-time session, single-trial connectivity between PMv and pSTS is continuously estimated and the plasticity readout is evaluated in the adaptive gating model. AI was used to generate some portions of the figures. The authors have reviewed and edited the output and take full responsibility for the content of this publication.

Importantly, this state-triggered implementation is not outcome-adaptive: the rule defining the candidate pathway state is specified a priori and is not modified according to the physiological consequences of preceding ccPAS volleys. Its numerical calibration may nevertheless be reassessed between blocks if predefined stationarity criteria indicate substantial drift in the underlying connectivity distribution. A more ambitious extension, which we outline as a direction for future development, would close the loop not only on pathway state but on the plasticity that stimulation *induces*—an *adaptive, outcome-based network-state-dependent ccPAS*. In such a scheme, the controller would progressively learn a subject-specific mapping between the pathway state at the time of stimulation and the ensuing physiological change, refining online its own definition of a plasticity-permissive state—an approach naturally connected to network control theory (see Figure 3). Unlike conventional closed-loop stimulation paradigms, such a controller would not drive functional connectivity toward a predefined target value: because the relationship between pathway state and stimulation efficacy is unknown and should not be assumed a priori, the state-plasticity mapping itself would be the object of learning rather than an assumption embedded in the control strategy. This step, however, faces a specific and as yet unsolved obstacle: ccPAS aftereffects accumulate gradually over many paired pulses and are conventionally quantified over minutes (e.g., [12]), so the effect of any single volley cannot presently be read out online. A sufficiently rapid and specific physiological feedback signal is therefore a prerequisite for, rather than a refinement of, this outcome-adaptive extension: without such a readout, the state–plasticity mapping cannot be learned or updated online. Whether a sufficiently sensitive and rapid surrogate of induced plasticity exists—for instance, intermittent dual-coil TMS probes of effective connectivity or TMS-EEG markers of potentiation interleaved with stimulation—remains an open question that will determine the feasibility of outcome-based, as opposed to state-triggered, adaptive ccPAS.

Across these stages, connectivity ceases to be a descriptive or post hoc outcome measure and becomes an endogenous physiological descriptor of pathway state that guides stimulation: first to personalize targets, then to trigger delivery according to the instantaneous pathway state, and, as a longer-term goal, to learn the state-plasticity relationship itself. We believe that this conceptual shift, from using connectivity to describe brain networks to using it to guide individualized neuromodulation, constitutes the central argument of the network-state-dependent framework proposed in this paper.

## 4. Technical and Methodological Considerations

Recent advances in EEG source imaging, computational neuroscience, and real-time signal processing make the *connectivity-informed* and *state-triggered* stages of the framework proposed here technically feasible using existing methodologies and technologies; the adaptive, outcome-based extension remains a longer-term goal (Section 3). What follows outlines the pipeline that these stages require and then turns to the methodological questions that will ultimately decide whether online pathway-state estimation is reliable enough to guide stimulation.

### 4.1. Anatomical Modeling

Subject-specific anatomical information derived from individual magnetic resonance images (MRI), together with accurate EEG electrode digitization, can be used to construct realistic head models and individualized leadfield matrices before the experimental session (Figure 2A). These computationally demanding steps need only be performed once, providing a stable anatomical framework for subsequent online analyses. While source-space definition and forward modeling can be performed days in advance with respect to the EEG-ccPAS session, the leadfield calculation needs the digitized positions of the individual’s EEG electrodes and must therefore be computed in the lab, at the start of the session but before ccPAS begins.

### 4.2. Resting-State Functional Connectivity Priors

Following the acquisition of a short resting-state EEG recording (typically 5–10 min), the data can be rapidly preprocessed and projected into source space using a beamforming approach such as Linearly Constrained Minimum Variance (LCMV) [119,120]. Source activity can then be summarized at the parcel level using an anatomical atlas, substantially reducing dimensionality while preserving the large-scale organization of cortical networks. Functional interactions between parcels may subsequently be quantified using leakage-robust connectivity metrics, such as the iPLV, which minimizes the influence of zero-lag correlations arising from volume conduction and residual source leakage [121,122,123]. In this way, both an individualized estimate of the baseline connectivity of the targeted pathway and subject-specific thresholds that define its candidate plasticity-permissive state are obtained.

### 4.3. Online Pathway-State Estimation and Triggering

During the subsequent ccPAS intervention, source activity within the targeted pathway is reconstructed online using the same individualized forward model and beamformer, enabling continuous estimation of pathway connectivity from successive sliding windows of neural activity. The estimated connectivity is repeatedly compared with the individualized threshold established during resting-state calibration and paired-pulse stimulation is delivered whenever the pathway is classified as occupying the target connectivity state. Importantly, connectivity is not an instantaneous variable, but a quantity estimated over a finite temporal window. Consequently, stimulation is contingent on the current estimate of pathway coupling rather than on the phase of an ongoing oscillation or the prediction of a discrete optimal stimulation instant.

Importantly, the objective here is not to estimate whole-brain connectivity continuously throughout stimulation. Rather, the primary goal of the initial resting-state recording is to obtain an accurate and individualized characterization of the subject’s resting-state network immediately before the experimental session. This network estimate can be computed within a few minutes and used to identify personalized stimulation targets, define the individualized thresholds, and set the network context for subsequent EEG-triggered ccPAS. During stimulation, computational requirements become substantially lighter, since only source activity within the targeted pathway and its corresponding connectivity state needs to be estimated online to determine whether the instantaneous pathway state satisfies the predefined rule. Crossing the predefined connectivity threshold constitutes a necessary but not sufficient condition for stimulation: a ccPAS volley is delivered only if the pathway-state criterion is satisfied, the predefined minimum inter-volley interval has elapsed, signal-quality criteria are met, and the predetermined maximum pulse count has not been reached.

### 4.4. Methodological Issues

Although this processing pipeline is now technically achievable, its successful implementation depends far more on methodological robustness than on computational speed. The principal challenge is to establish whether parcel-level source-space connectivity can be estimated with sufficient reliability, spatial specificity, and temporal resolution to support online decision-making within a single experimental session. In practice, the complete pipeline, including preprocessing, source reconstruction, connectivity estimation, and state classification, must operate within a time window compatible with state-triggered stimulation while maintaining stable physiological estimates.

Several methodological issues therefore become critical. Reliable estimation depends on accurate forward modeling, robust source reconstruction, appropriate atlas parcellation, and the selection of connectivity measures that remain resilient to source leakage, measurement noise, muscular artifacts, and inter-individual anatomical variability [124]. Leakage-insensitive metrics such as iPLV are particularly attractive because they reduce spurious zero-lag interactions, although their suitability for online applications remains to be systematically evaluated. Equally important is the reproducibility of pathway-state estimates across recording sessions [125]. A connectivity measure that performs well in retrospective offline analyses may not necessarily retain the same reliability when estimated online from a brief resting-state recording immediately preceding stimulation.

Importantly, source-space functional connectivity should not be interpreted as a direct measure of synaptic communication between the targeted regions [126]. Estimated coupling may partly reflect common network input, residual volume conduction/source leakage, or indirect polysynaptic interactions [122,124]. Moreover, although iPLV is phase-based, variations in local oscillatory power and signal-to-noise ratio may influence the reliability of phase and connectivity estimates. Leakage-resistant measures reduce specific effects of instantaneous signal mixing but do not resolve these broader ambiguities. Accordingly, interpretation of pathway state should remain at the level of functional interaction between the targeted populations rather than implying direct anatomical or monosynaptic communication.

A distinct issue concerns the directionality of the connectivity estimate. ccPAS is an inherently directional intervention, and reversing the order of the paired pulses yields dissociable, often opposite, effects (Box 1). Yet, phase-based coupling metrics such as the iPLV are undirected: they quantify the strength of coupling but not its direction, and may therefore provide an incomplete descriptor of a state intended to guide a direction-specific protocol [127]. Directed estimators—Granger-causality measures, the phase-slope index, or transfer entropy—would in principle be better matched to this logic, but are more demanding in data, model assumptions, and computation, and their online estimation within a triggering loop has not been established [127,128]. Whether an undirected estimate of coupling strength suffices to define a plasticity-permissive state, or whether directional information is required, is itself an empirical question raised by the framework.

An additional consideration is that connectivity-defined pathway states may covary with broader fluctuations in physiological and behavioral state, including arousal, vigilance, attention, and muscle activity. Because these factors can themselves influence cortical excitability and susceptibility to plasticity, an apparent relationship between pathway connectivity and ccPAS efficacy cannot automatically be attributed to the targeted cortico-cortical interaction. Initial validation experiments should therefore monitor relevant complementary variables, such as pupil diameter or subjective vigilance, muscle activity through EMG, and local EEG features including oscillatory power. These measures can be incorporated into control analyses to determine whether connectivity-defined pathway state explains variability in stimulation-induced plasticity beyond that accounted for by broader physiological-state fluctuations. Importantly, this does not require pathway connectivity to be independent of global brain state; rather, it tests whether it provides additional information relevant to plasticity beyond these potential confounds.

An additional challenge concerns the temporal scale over which pathway state should be estimated. Unlike oscillatory phase, which can be determined from individual cycles, functional connectivity generally requires temporal integration across multiple oscillatory cycles to achieve robust estimation, and this has important implications for real-time applications. First, the integration window introduces an intrinsic latency between a given physiological event and the availability of its corresponding connectivity estimate. Together with the computational time required for preprocessing, source reconstruction, and connectivity estimation, this latency defines the temporal precision with which stimulation can be aligned to a target pathway state and should therefore be explicitly quantified for any candidate real-time pipeline. Second, because connectivity is estimated over finite temporal windows rather than sampled instantaneously, the dynamics of pathway states remain incompletely understood [129]. An important open question is whether connectivity states persist sufficiently long to permit reliable online detection and stimulation before transitions occur. Accordingly, a necessary feasibility condition for real-time pathway-state triggering is that the characteristic dwell time of the candidate state be sufficiently long relative to the effective temporal lag of its estimation, processing, and trigger delivery. This relationship should be quantified empirically rather than assumed. Characterizing state stability, dwell-time distributions, and transition dynamics will therefore be essential: if states change more rapidly than they can be reliably detected and acted upon, that connectivity metric, frequency band, or state definition would not constitute a viable triggering variable [130]. These properties will ultimately determine the appropriate balance between estimation-window duration, detection latency, and stimulation timing.

Unlike local oscillatory phases, functional connectivity metrics are not intrinsically periodic variables. Rather than defining a rhythmic target, they quantify the ongoing interaction between distributed neural populations over a finite temporal window. Consequently, network-state-dependent stimulation does not require interareal synchrony to fluctuate cyclically. Instead, it relies on detecting transient connectivity states that emerge irregularly over time but recur with sufficient frequency to allow reliable online detection once an appropriate decision threshold has been established [130,131]. An important practical consideration is that this threshold must be carefully calibrated: if set too low, stimulation may be triggered excessively, whereas an overly stringent threshold may yield trigger events that are too infrequent for efficient protocol execution. This challenge further highlights the value of individualized offline calibration, whereby resting-state recordings can be used to characterize the distribution of the selected connectivity metric and determine subject-specific thresholds before the real-time experiment, ensuring both physiologically meaningful and experimentally feasible trigger rates. For an initial experimental implementation, candidate high- and low-connectivity states could be defined using subject-specific percentiles of the connectivity distribution obtained during the pre-stimulation resting-state recording (e.g., values above the 75th and below the 25th percentile, respectively). Percentile-based thresholds provide a transparent, scale-independent starting point but do not uniquely determine the online trigger rate, which will additionally depend on the temporal autocorrelation and dwell-time distribution of the connectivity state, and on protocol constraints such as the minimum inter-volley interval. Threshold selection should therefore be regarded as a control parameter balancing physiological state selectivity against the availability of sufficient trigger opportunities within an experimentally viable session duration. Overall, this approach represents a conceptual shift from phase-triggered stimulation, in which the control variable is inherently oscillatory, to network-state-triggered stimulation, in which the control variable is the evolving interaction between cortical regions, irrespective of whether it follows a periodic temporal structure.

Concurrent TMS introduces an additional constraint on online pathway-state estimation. Although the connectivity estimate triggering a given volley is computed from pre-stimulation EEG, the TMS pulse itself produces a large electromagnetic artifact that may transiently saturate the EEG amplifier and is followed by electrode, muscle, auditory, and movement-related contamination [132,133]. Connectivity estimation should therefore be suspended for a predefined post-stimulation exclusion interval and resumed only after amplifier recovery and predefined signal-quality criteria are satisfied [133,134]. Windows containing excessive amplitude, muscle activity, movement, or other artifacts should be classified as ineligible for triggering rather than used for connectivity estimation [134]. The appropriate exclusion interval must be empirically determined for the specific hardware, stimulation parameters, cortical targets, and online processing pipeline [132,135].

A related consideration concerns the within-session stability of the connectivity distribution used for threshold calibration. Although pathway state is expected to fluctuate over time, the baseline distribution from which the triggering threshold is derived cannot necessarily be assumed to remain stationary throughout the stimulation session. In an initial implementation, threshold stability could therefore be assessed across stimulation blocks using brief stimulation-free EEG periods. If substantial distributional drift is detected, recalibration could be performed between blocks using the same predefined decision rule. Such recalibration would preserve the a priori definition of the candidate pathway state while accounting for slow changes in the underlying connectivity distribution, and is conceptually distinct from outcome-adaptive updating of the stimulation rule.

Taken together, these considerations define the primary challenge for network-state-dependent ccPAS: it is not whether source-space connectivity can be computed rapidly enough, but whether it can be estimated accurately, reproducibly, and with sufficient physiological validity to serve as a reliable descriptor of pathway state. Establishing this reliability—including end-to-end estimation latency and temporal jitter, robustness to physiological and non-physiological artifacts, test–retest reproducibility, and agreement between offline and online estimates—constitutes a necessary methodological milestone before source-space functional connectivity can be responsibly adopted as an endogenous variable for network-state-dependent neuromodulation. In this framework, pathway-state estimation and stimulation direction constitute two distinct control dimensions: the former determines when ccPAS is delivered, whereas pulse order and inter-stimulus interval determine the direction in which the intervention is intended to modify the pathway. Whether these dimensions are physiologically separable is unknown: endogenous directionality may carry information about plasticity susceptibility that is not captured by undirected coupling strength. Accordingly, the adequacy of iPLV as a state descriptor, and the potential added value of directed connectivity estimates, should be determined empirically. Box 2 summarizes the key methodological considerations for choosing the appropriate connectivity metric.

An important complementary validation strategy may be provided by studies combining TMS with intracranial EEG (iEEG), ideally together with simultaneous scalp EEG [136,137]. Although the present framework focuses on scalp EEG because its intended implementation is non-invasive, intracranial recordings could provide a valuable reference for evaluating the physiological validity of source-reconstructed pathway-state estimates [138]. Simultaneous iEEG and scalp EEG could test whether connectivity states identified non-invasively correspond to interregional coupling observed intracranially, while also providing higher-resolution estimates of their temporal stability, dwell times, and transition dynamics. Such recordings could further help disentangle genuine interregional interactions from residual signal mixing, common input, and indirect network effects. Their interpretation would nevertheless remain constrained by the sparse and clinically determined spatial sampling of iEEG [139]. We therefore regard combined TMS–iEEG–scalp EEG studies as a complementary validation platform rather than as an alternative to the non-invasive implementation proposed here.

Box 2 **Connectivity metric selection: balancing physiological relevance and robustness to source leakage.**

 A critical methodological consideration for network-state-dependent neuromodulation is the selection of an appropriate functional connectivity metric. Conventional phase-based measures, such as the phase-locking value (PLV), are sensitive to source leakage and volume conduction, which can introduce spurious correlations between neighboring cortical regions after source reconstruction [62,121,122,123]. Nevertheless, comparisons with intracranial recordings suggest that non-invasively estimated phase synchronization can still capture physiologically meaningful patterns of interregional interactions despite these systematic biases [140]. To mitigate the effects of source leakage, leakage-resistant metrics such as the imaginary part of the phase-locking value (iPLV) have been proposed, as they selectively quantify non-zero-lag phase synchronization while suppressing interactions that are likely to arise from instantaneous mixing. Although this approach inevitably sacrifices sensitivity to genuine zero-lag coupling, it provides a practical compromise between physiological specificity and robustness [123]. For adaptive brain-state-dependent stimulation, where connectivity estimates directly influence stimulation decisions, minimizing false-positive estimates of network synchronization may be more important than maximizing sensitivity to every possible interaction. Accordingly, the rationale for adopting iPLV is not to replace an otherwise invalid measure, but rather to improve the specificity of phase-based connectivity estimates in source-space analyses.

 Methodological comparisons further suggest that iPLV offers a favorable balance between robustness to signal mixing, reliable estimation of phase synchronization, and computational suitability for real-time implementation [141].

 The practical feasibility of this approach has recently been demonstrated in real-time applications. Ref. [53] showed that EEG-guided TMS can be triggered using interhemispheric functional connectivity estimated from ongoing EEG and subsequently employed iPLV to verify that the observed effects were not solely attributable to volume conduction. Building on this work, Ref. [142] implemented source-space, network-state-dependent TMS using μ-band iPLV to characterize connectivity within the human motor network, providing a proof of concept for adaptive stimulation based on ongoing large-scale cortical interactions.

 Together, these studies demonstrate that leakage-resistant, phase-based connectivity measures can be implemented online and integrated into closed-loop stimulation paradigms. Importantly, no functional connectivity metric has yet emerged as a universally optimal solution for source-space EEG/MEG analyses [124,125]. Recent validation against simultaneous intracranial recordings has highlighted that different source-space connectivity metrics vary considerably in their correspondence with intracranial measures, emphasizing that metric selection is a critical methodological consideration [140]. These findings indicate that connectivity estimation remains one of the principal methodological challenges in source-space electrophysiology and emphasize that metric selection should be guided by the specific requirements of the intended application rather than by theoretical considerations alone. For real-time source-space network estimation, where robustness, computational efficiency, and physiological interpretability must be carefully balanced, iPLV currently represents a well-justified and pragmatic choice. However, the framework proposed here is intentionally independent of the specific connectivity estimator employed. Any metric that can be estimated online with sufficient robustness, temporal resolution, and physiological validity could, in principle, serve as the endogenous descriptor of pathway state.

## 5. Biological Plausibility and Open Questions

The framework proposed in this paper rests on the hypothesis that the online connectivity estimate of a targeted cortico-cortical pathway represents a biologically meaningful descriptor of its physiological state and may therefore predict its susceptibility to stimulation-induced plasticity. At present, however, this should be regarded as a plausible and experimentally testable hypothesis rather than an established principle. Although substantial evidence demonstrates that the effects of non-invasive brain stimulation depend on the ongoing brain state, considerably less is known about whether the instantaneous state of communication between interconnected cortical regions directly influences the efficacy of plasticity-inducing interventions such as ccPAS.

Several complementary lines of evidence support the biological plausibility of this hypothesis. First, a growing body of work has demonstrated that the induction of cortical plasticity is strongly state-dependent, with stimulation outcomes varying according to oscillatory phase, oscillatory power, and measures of inter-regional synchronization [53,55,142,143]. Second, ccPAS is generally interpreted within a Hebbian-like STDP framework [2,3], whereby repeated and appropriately timed activation of interconnected neuronal populations strengthens their functional interactions. Finally, theories of communication-through-coherence propose that transient synchronization between distributed cortical regions dynamically regulates information transfer, suggesting that periods of enhanced functional coupling may also correspond to periods of increased susceptibility to plasticity-inducing stimulation. Together, these observations provide a coherent theoretical rationale for considering the functional connectivity of a targeted pathway as a candidate descriptor of its instantaneous physiological state.

At the same time, several neurophysiological considerations caution against assuming a simple relationship between pathway connectivity and stimulation-induced plasticity. Beyond Hebbian mechanisms, theories of metaplasticity such as the Bienenstock–Cooper–Munro (BCM) framework propose that the threshold for inducing synaptic potentiation is itself activity-dependent, shifting according to the recent history of neuronal activation. From this perspective, the physiological state of a pathway may determine not only whether plasticity can be induced, but also the conditions under which potentiation or depression become more likely [144,145]. Homeostatic plasticity mechanisms may oppose additional strengthening when networks are already highly synchronized, leading to saturation or ceiling effects [146,147]. Likewise, the dependence of plasticity on pathway state may be nonlinear, following an inverted-U relationship in which maximal susceptibility occurs at intermediate rather than maximal levels of functional connectivity. The optimal pathway state may further depend on the cortical system under investigation, oscillatory frequency, behavioral context, or individual neurophysiological characteristics. Consequently, it cannot be assumed that greater functional connectivity necessarily predicts greater plasticity. These uncertainties have a direct experimental corollary: a first test of the framework need not assume the shape of this relationship, but could contrast ccPAS delivered during high-connectivity, low-connectivity, and randomly timed states, with “high” and “low” defined by the individualized thresholds derived from each participant’s initial resting-state recording, thereby establishing *whether* pathway state modulates the induced plasticity before asking *how*.

Accordingly, we do not propose that pathway connectivity should already be adopted as a validated control variable for neuromodulation. Rather, we argue that it should become the principal experimental variable through which the relationship between pathway state and stimulation-induced plasticity is systematically investigated. The central scientific question is therefore not whether ccPAS modifies functional connectivity, which several studies suggest it can [11,38,61,148,149], but whether the online connectivity estimate of the targeted pathway before and during stimulation predicts the magnitude, direction, or reproducibility of the induced plastic changes.

Addressing this question defines a critical frontier for network-state-dependent neuromodulation. The fundamental unknown is the exact mathematical relationship between pre-stimulation pathway state and induced plasticity. Does plasticity increase monotonically with functional connectivity, saturate past a threshold, or follow an inverted-U trajectory? Furthermore, it remains to be established whether these dynamics vary predictably across different cortical systems and oscillatory frequencies, or if they represent highly individual features that can only be captured through real-time optimization—the aspiration of the adaptive, outcome-based extension outlined above.

A further open question concerns the translational value of this framework. A principal motivation for network-state-dependent ccPAS is its potential to mitigate the considerable inter-individual variability that currently limits the reproducibility of paired associative stimulation [12,92]. Whether stimulation strategies guided by individualized, real-time estimates of pathway state ultimately prove more reliable than conventional protocols, such as those based purely on anatomical targeting or local oscillatory features, remains an empirical question rather than a foregone conclusion.

From this perspective, network-state-dependent stimulation is not a tool for forcing functional connectivity toward a predefined target value. Rather, it is an experimental framework for mapping, on an individual basis, the hidden physiological transfer function between pathway state and plasticity. Only by first charting this relationship can source-space functional connectivity be responsibly adopted as a genuine endogenous control variable for adaptive neuromodulation.

## 6. Future Perspectives: Toward Personalized Network Neuromodulation

The framework proposed in this opinion paper should not be viewed as a finalized stimulation protocol, but rather as the foundation of a broader research agenda aimed at redefining how endogenous brain states are characterized in adaptive neuromodulation. The recent convergence of advances in EEG source imaging, computational neuroscience, and real-time signal processing has made it technically feasible to estimate the functional state of cortico-cortical pathways within the timescale of a single experimental session. Yet, as discussed above, technical feasibility is only a precondition: whether such estimates are reliable and reproducible enough to guide stimulation remains an open methodological question, and beyond it lies a deeper biological one: whether the instantaneous physiological state of a pathway predicts its susceptibility to stimulation-induced plasticity.

Addressing this question has implications that extend far beyond ccPAS itself. More broadly, it challenges the traditional paradigm that adaptive neuromodulation must be guided exclusively by local electrophysiological variables, such as oscillatory phase or power. We propose instead that the physiological state of distributed cortical pathways constitutes a more appropriate endogenous variable for interventions designed specifically to modify communication between brain regions. In its most ambitious, outcome-based form, such an approach would let stimulation move from a protocol reacting to ongoing neural activity toward one that progressively learns the individualized relationship between pathway state and plasticity, transforming stimulation from a static paradigm into a continuously evolving experimental strategy that refines itself as it proceeds.

From a clinical standpoint, this approach directly addresses a principal limitation of contemporary non-invasive brain stimulation: the substantial inter-individual variability that hampers reproducibility and limits therapeutic efficacy [150,151,152,153,154]. Current protocols largely rely on anatomical localization or local electrophysiological markers that may only partially capture the network-level conditions required to induce plasticity. ccPAS has already begun to be explored in disorders characterized by network dysfunction—including visual processing deficits after stroke [155,156], Alzheimer’s disease [157], schizophrenia [158], chronic alcoholism [159], and, more broadly, psychiatric conditions in which maladaptive interregional interactions have been targeted or proposed as potential targets for pathway-specific neuromodulation (for a review, see [2]; see also [160,161,162,163,164,165]). In these populations, altered or absent after-effects highlight precisely the responsiveness differences that a state-guided approach aims to address. Indeed, in stroke, ccPAS effects have been found to depend on individual pathway integrity and functional coupling, with clear responders and non-responders [155]. If pathway-state estimation proves predictive of stimulation efficacy, tailoring the timing of stimulation to each subject’s network state could improve consistency across repeated sessions and inform patient stratification.

Achieving this vision will require coordinated, systematic methodological efforts across the brain stimulation community. Standardized source-reconstruction pipelines, leakage-robust connectivity estimators, harmonized validation procedures, and shared benchmark datasets will be essential to establish the reliability and reproducibility of online pathway-state estimation across different laboratories. Equally critical will be prospective studies designed with a dual purpose: not merely to evaluate whether connectivity changes *after* stimulation, but to determine whether the instantaneous state of a targeted pathway *predicts* the plasticity that follows, and whether this relationship can be mapped within a single experimental session.

Ultimately, we envision a profound conceptual shift in how functional connectivity is used in neuromodulation. Rather than a descriptive, retrospective quantity computed after an experiment has concluded, it could become an endogenous physiological control variable that informs experimental design and guides stimulation delivery in real time. Demonstrating that pathway-state-guided stimulation improves the reproducibility and efficacy of neuromodulation would represent a major milestone for precision brain stimulation—tailored not only to the static anatomy of the individual brain, but to its living, continuously evolving network state.

In our view, the next generation of brain stimulation will be personalized not simply because it targets the right cortical region, but because it intervenes when the targeted cortico-cortical pathway is in the physiological state that is most permissive for plasticity.

## Data Availability

The original contributions presented in this study are included in the article. Further inquiries can be directed to the corresponding author.

## Abbreviations

The following abbreviations are used in this manuscript:

AON	Action-Observation Network
ccPAS	Cortico-Cortical Paired Associative Stimulation
EEG	Electroencephalography
fMRI	Functional Magnetic Resonance Imaging
iEEG	Intracranial EEG
iPLV	Imaginary part of the Phase Locking Value
LCMV	Linearly Constrained Minimum Variance
LTD	Long-Term Depression
LTP	Long-Term Potentiation
MRI	Magnetic Resonance Imaging
NIBS	Non-Invasive Brain Stimulation
PLV	Phase-Locking Value
STDP	Spike-Timing-Dependent Plasticity
TMS	Transcranial Magnetic Stimulation
